# Heterogeneous benefit of early sacubitril/valsartan initiation after PCI in elderly patients with left ventricular dysfunction: myocardial recovery and mid-term cardiovascular outcomes

**DOI:** 10.3389/fcvm.2026.1843561

**Published:** 2026-05-29

**Authors:** Jian Li, Chunxia Tang

**Affiliations:** Wuxi People's Hospital Affiliated to Nanjing Medical University, Wuxi, China

**Keywords:** elderly patients, left ventricular dysfunction, major adverse cardiovascular events, myocardial recovery, percutaneous coronary intervention, sacubitril/valsartan

## Abstract

**Background:**

The optimal timing of sacubitril/valsartan initiation after percutaneous coronary intervention (PCI) in elderly patients with left ventricular dysfunction remains unclear. This study evaluated whether very early initiation was associated with better myocardial recovery and clinical outcomes than delayed initiation or no sacubitril/valsartan.

**Methods:**

In this single-center retrospective cohort study, elderly patients with left ventricular dysfunction after successful PCI were classified into three groups according to treatment timing: early initiation (≤7 days), delayed initiation (14–28 days), and no sacubitril/valsartan. After 1:1:1 propensity score matching, 360 patients were included. Echocardiographic recovery and biomarker changes were assessed at 8 weeks, and major adverse cardiovascular events (MACE) were evaluated over 12 months using competing-risk analysis.

**Results:**

In the matched cohort, the early initiation group showed the greatest improvement in left ventricular ejection fraction and cardiac index, together with the largest reductions in left ventricular dimensions at 8 weeks. Early initiation was also associated with more favorable declines in inflammatory and cardiac injury biomarkers, including hs-CRP and NT-proBNP. During 12-month follow-up, MACE occurred in 8.3% of the early group, 20.8% of the delayed group, and 25.0% of the control group. In Fine-Gray models, early initiation was associated with a significantly lower risk of MACE than delayed initiation and no sacubitril/valsartan. Exploratory subgroup analyses suggested stronger associations in patients aged ≥75 years and those with baseline LVEF ≤40%. Safety outcomes were comparable across groups.

**Conclusions:**

In this propensity score-matched retrospective analysis of elderly patients with left ventricular dysfunction after PCI, initiation of sacubitril/valsartan within 7 days was associated with more favorable short-term myocardial recovery profiles and a lower 12-month cardiovascular risk compared with delayed initiation or no ARNI therapy. However, residual confounding and potential immortal time bias cannot be excluded. These hypothesis-generating findings suggest a potential association that merits evaluation in prospective, randomized studies designed to compare different treatment timing strategies.

## 1 Introduction

Percutaneous coronary intervention (PCI) has become the cornerstone of revascularization in patients with acute coronary syndrome (ACS), substantially reducing early mortality (1, 2). However, in elderly patients—particularly those with pre-existing left ventricular dysfunction—the post-PCI course remains frequently complicated by persistent impairment of myocardial contractility, sustained inflammatory activation, and an increased risk of subsequent cardiovascular events (3, 4). Even after technically successful revascularization, the degree of myocardial recovery varies considerably, with a substantial proportion of patients failing to achieve meaningful functional improvement (5). This heterogeneity underscores the clinical importance of optimizing post-PCI pharmacological strategies, particularly with regard to the timing of initiation of disease-modifying therapies.

Sacubitril/valsartan, a first-in-class angiotensin receptor–neprilysin inhibitor (ARNI), has transformed the management of heart failure with reduced ejection fraction (HFrEF), demonstrating superiority over enalapril in reducing cardiovascular mortality and heart failure hospitalization in the PARADIGM-HF trial (6, 7). Beyond its established role in chronic heart failure, accumulating evidence suggests that sacubitril/valsartan may also exert beneficial effects on cardiac remodeling and myocardial recovery in the post–myocardial infarction setting. For example, Zhang et al. (8) reported that, among patients with ST-segment elevation myocardial infarction (STEMI), early administration of sacubitril/valsartan within 24 h after primary PCI was associated with improved left ventricular ejection fraction (LVEF) and lower NT-proBNP levels compared with conventional ACEI therapy. Similarly, Liu et al. (9) found that in ACS patients with reduced LVEF after PCI, sacubitril/valsartan use was associated with improvements in LVEF, reductions in NT-proBNP, and attenuation of left ventricular remodeling at six months. In addition, meta-analyses by Zhang et al. (10) and Zhao et al. (11) suggested that early initiation of sacubitril/valsartan after acute myocardial infarction may be associated with improved cardiac function and a lower incidence of adverse cardiovascular events.

Despite these encouraging findings, important knowledge gaps remain. First, the optimal timing of sacubitril/valsartan initiation after PCI—particularly the relative effects of very early vs. delayed initiation—has not been well defined in elderly patients with left ventricular dysfunction. Second, most prior studies have focused on average treatment effects, with limited evaluation of whether certain patient subgroups may derive differential benefit from earlier initiation (12). Given that older patients often exhibit distinct pathophysiological features, including heightened inflammatory responses, increased susceptibility to adverse ventricular remodeling, and higher baseline event risk (13, 14), understanding potential heterogeneity in treatment response is particularly relevant in this population.

Accordingly, several clinically relevant questions remain insufficiently addressed. First, whether very early initiation of sacubitril/valsartan after PCI is associated with incremental benefit compared with delayed initiation or no ARNI therapy in elderly patients with left ventricular dysfunction. Second, whether differences in treatment timing are associated with distinct patterns of myocardial recovery and biomarker trajectories. Third, whether any observed associations are more pronounced in clinically vulnerable subgroups, such as patients of advanced age or those with more severe baseline systolic dysfunction (15–17).

Therefore, the present study was designed not only to evaluate the potential association between sacubitril/valsartan use and post-PCI outcomes, but also to examine whether the timing of initiation is related to differences in myocardial recovery, biomarker changes, and subsequent cardiovascular risk. Specifically, we compared very early initiation (≤7 days), delayed initiation (14–28 days), and no sacubitril/valsartan, with the exploratory aim of characterizing timing-related differences. and of investigating whether these associations vary across clinically relevant subgroups. Given the observational design, all findings are intended to be hypothesis-generating rather than confirmatory.

## 2 Methods

### 2.1 Study design and setting

This was a single-center, retrospective cohort study conducted at Wuxi People's Hospital between March 2023 and June 2025. The study was designed to evaluate whether the timing of sacubitril/valsartan initiation after successful PCI was associated with differences in short-term myocardial recovery, biomarker trajectories, and 12-month cardiovascular outcomes in elderly patients with left ventricular dysfunction. The study protocol was approved by the institutional review board of Wuxi People's Hospital, and the requirement for written informed consent was waived because of the retrospective nature of the study. All procedures were performed in accordance with the Declaration of Helsinki.

### 2.2 Study population

Consecutive patients aged 65 years or older who underwent successful PCI for acute coronary syndrome or stable coronary artery disease during the study period were screened. To be eligible, patients were required to have documented left ventricular systolic dysfunction, defined as a LVEF of 45% or less on transthoracic echocardiography performed within 7 days after the index PCI. The date of PCI was defined as the index date for cohort entry and subsequent follow-up.

The index date (time zero) for all outcome analyses was defined as the date of successful PCI. This fixed time point ensures that exposure assessment (particularly for the delayed initiation group, who started therapy between days 14–28) is not subject to immortal time bias arising from the requirement to survive free of events until treatment initiation.

Patients were excluded if they met any of the following criteria: (1) incomplete echocardiographic or biomarker data at the planned 8-week follow-up; (2) contraindications to sacubitril/valsartan, including a history of angioedema, severe hepatic impairment, or estimated glomerular filtration rate (eGFR) <30 mL/min/1.73 m²; (3) prior use of sacubitril/valsartan within 6 months before the index PCI; (4) severe valvular heart disease requiring intervention; or (5) expected survival <12 months because of non-cardiovascular comorbidity. After screening 512 patients, 406 fulfilled the eligibility criteria and entered the unmatched cohort.

### 2.3 Exposure definition and treatment groups

Patients were categorized into three mutually exclusive groups according to the timing of sacubitril/valsartan initiation after the index PCI. The Early Initiation group included patients who started sacubitril/valsartan within 7 days after PCI. The Delayed Initiation group included patients who started sacubitril/valsartan between 14 and 28 days after PCI. The Control group included patients who did not receive sacubitril/valsartan during the study observation period.

Exposure classification was based on inpatient medication records, discharge prescriptions, and follow-up treatment documentation in the electronic medical record. In the Early and Delayed groups, sacubitril/valsartan was prescribed according to routine clinical practice, and patients previously receiving an angiotensin-converting enzyme inhibitor (ACEI) underwent a 36 h washout period before initiation, whereas those receiving an angiotensin receptor blocker (ARB) were switched directly when clinically appropriate. Background therapy, including dual antiplatelet therapy, statins, beta-blockers, and other guideline-directed cardiovascular medications, was prescribed at the discretion of the treating physicians in accordance with contemporary practice. Patients in the Control group could receive conventional ACEI/ARB-based therapy but did not receive sacubitril/valsartan within the prespecified exposure window.

### 2.4 Baseline assessment and data collection

Baseline data were obtained from the hospital electronic medical record system. Demographic variables included age, sex, and body mass index (BMI). Clinical covariates included hypertension, diabetes mellitus, dyslipidemia, prior myocardial infarction, prior heart failure, atrial fibrillation, and chronic kidney disease, defined as eGFR <60 mL/min/1.73 m². Information on discharge medications, including aspirin, P2Y12 inhibitors, beta-blockers, ACEI/ARB, and statins, was also collected.

Baseline echocardiographic assessment included LVEF, left ventricular end-diastolic diameter (LVEDD), left ventricular end-systolic diameter (LVESD), and cardiac index (CI). Baseline laboratory evaluation included inflammatory biomarkers, myocardial injury markers, and neurohormonal markers measured from fasting venous blood samples obtained during the index hospitalization before or shortly after treatment allocation according to routine care.

### 2.5 Propensity score matching

Because treatment allocation was not randomized, propensity score matching (PSM) was used to reduce baseline imbalance and confounding by indication. A 1:1:1 matched cohort was generated across the Control, Delayed Initiation, and Early Initiation groups using multinomial logistic regression to estimate generalized propensity scores. The prespecified covariates included age, sex, BMI, hypertension, diabetes mellitus, dyslipidemia, prior myocardial infarction, prior heart failure, atrial fibrillation, chronic kidney disease, baseline LVEF, baseline LVEDD, and baseline use of beta-blockers, ACEI/ARB, and statins.

Nearest-neighbor matching was performed with a caliper width of 0.2 times the standard deviation of the logit-transformed propensity score. Matching was intended to retain patients with comparable baseline clinical risk profiles across all three treatment strategies. Covariate balance before and after matching was assessed using standardized mean differences (SMDs), with an SMD <0.10 considered indicative of acceptable balance.

Missing data for any covariate (present in <2% of screened patients) were handled by complete-case analysis before matching, given the low proportion of missingness. Post-matching balance diagnostics confirmed excellent balance for all covariates except ACEi/ARB use, which remained intentionally imbalanced as it was a direct consequence of the exposure definition (early ARNI initiation precludes concurrent ACEi/ARB therapy). The SMD for ACEi/ARB use after matching was 1.28, reflecting this structural difference.

After matching, 360 patients remained in the analytic cohort, including 120 patients in each group. The covariate balance profile was additionally illustrated using a love plot.

### 2.6 Outcome measures

#### 2.6.1 Echocardiographic myocardial recovery outcomes

The primary mechanistic outcomes were changes in echocardiographic indices from baseline to 8-week follow-up, including LVEF, LVEDD, LVESD, and CI. The baseline echocardiographic examination was defined as the assessment performed within 7 days after the index PCI, and the follow-up examination was the routine reassessment performed approximately 8 weeks later. Absolute changes were calculated as follow-up minus baseline values for each parameter. Echocardiographic examinations were performed by experienced cardiologists using standardized institutional protocols.

#### 2.6.2 Clinical outcome

The primary clinical outcome was major adverse cardiovascular events (MACE) within 12 months after the index PCI. MACE was defined as a composite of cardiovascular death, non-fatal myocardial infarction, unplanned revascularization, hospitalization for heart failure, or stroke. Time-to-event follow-up was calculated from the date of the index PCI. For patients with more than one event, the first qualifying event was used in the primary time-to-event analysis. Non-cardiovascular death was treated as a competing event in the primary clinical outcome analysis.

#### 2.6.3 Biomarker outcomes

Secondary mechanistic outcomes included changes in inflammatory, myocardial injury, and neurohormonal biomarkers between baseline and 8 weeks. Inflammatory biomarkers included high-sensitivity C-reactive protein (hs-CRP), interleukin-6 (IL-6), tumor necrosis factor-alpha (TNF-α), and intercellular adhesion molecule-1 (ICAM-1). Cardiac injury and neurohormonal biomarkers included N-terminal pro-B-type natriuretic peptide (NT-proBNP), cardiac troponin I (cTnI), creatine kinase-MB (CK-MB), and lactate dehydrogenase (LDH). All assays were performed in the central laboratory of the study hospital using standardized commercial methods. For biomarker analyses, percentage change from baseline was calculated to facilitate comparison across markers with different absolute scales.

#### 2.6.4 Safety outcomes

Safety outcomes included symptomatic hypotension, renal impairment, bradycardia, hyperkalemia, and angioedema. Renal impairment was defined as an increase in serum creatinine of at least 50% from baseline. Bradycardia was defined as heart rate <50 beats/min, and hyperkalemia was defined as serum potassium >5.5 mmol/L. Safety events were identified from inpatient records, outpatient follow-up records, and laboratory data during the observation period.

### 2.7 Subgroup analysis

To explore whether the association between treatment timing and clinical outcome varied across clinically relevant subgroups, prespecified subgroup analyses were performed according to age (<75 vs. ≥75 years), sex, diabetes mellitus status, and baseline LVEF (≤40% vs. >40%). These subgroup categories were selected because they represented clinically plausible modifiers of post-PCI recovery and cardiovascular risk in elderly patients with left ventricular dysfunction. Interaction testing was performed to examine whether the association between treatment timing and MACE differed across subgroup strata.

### 2.8 Statistical analysis

Continuous variables were summarized as mean ± standard deviation (SD) when approximately normally distributed, or as median with interquartile range (IQR) when non-normally distributed. Categorical variables were presented as counts and percentages. Baseline characteristics were compared among groups using one-way analysis of variance (ANOVA) or the Kruskal–Wallis test for continuous variables, and the chi-square test or Fisher's exact test for categorical variables, as appropriate.

For echocardiographic outcomes, analysis of covariance (ANCOVA) was used to compare 8-week changes among groups with adjustment for baseline values of the corresponding echocardiographic parameter. When the overall group comparison was significant, pairwise comparisons were performed with Bonferroni correction. Biomarker changes were analyzed as percentage changes from baseline and compared across groups using the Kruskal–Wallis test, followed by Bonferroni-adjusted pairwise testing where appropriate.

For the primary clinical outcome, cumulative incidence curves were estimated using the cumulative incidence function to account for the competing risk of non-cardiovascular death, and group differences were assessed using Gray's test. Fine-Gray subdistribution hazard models were fitted to estimate subdistribution hazard ratios (SHRs) and 95% confidence intervals (CIs) for the association between treatment timing and 12-month MACE. Subgroup analyses were also performed within the Fine-Gray framework, with interaction terms used to assess potential heterogeneity of association across subgroup strata.

To assess the potential impact of immortal time bias (where patients in the delayed group must survive event-free until day 14 to be classified as such), two sensitivity analyses were planned: (1) a 14-day landmark analysis, where patients experiencing MACE or non-cardiovascular death within the first 14 days were excluded, and (2) a time-dependent Cox model treating sacubitril/valsartan initiation as a time-varying exposure. Given the exploratory nature of the study, no adjustment for multiple comparisons was applied to secondary or exploratory outcomes, and *P* values should be interpreted descriptively.

All statistical tests were two-sided, and a *P* value < 0.05 was considered statistically significant unless multiple pairwise comparisons required Bonferroni adjustment. Statistical analyses were performed using R version 4.2.1 (R Foundation for Statistical Computing, Vienna, Austria), primarily with the cmprsk, survival, and tidyverse packages.

## 3 Results

The results are organized according to prespecified outcome hierarchy. The primary clinical outcome was 12-month MACE analyzed with competing risks. Secondary mechanistic outcomes included 8-week changes in echocardiographic parameters (LVEF, LVEDD, LVESD, cardiac index) and key biomarkers (hs-CRP and NT-proBNP as representative markers). Exploratory outcomes comprised changes in other inflammatory and myocardial injury biomarkers (IL-6, TNF-α, ICAM-1, cTnI, CK-MB, LDH), subgroup analyses, and safety events.

### 3.1 Cohort assembly and matched population

**Figure 1:**
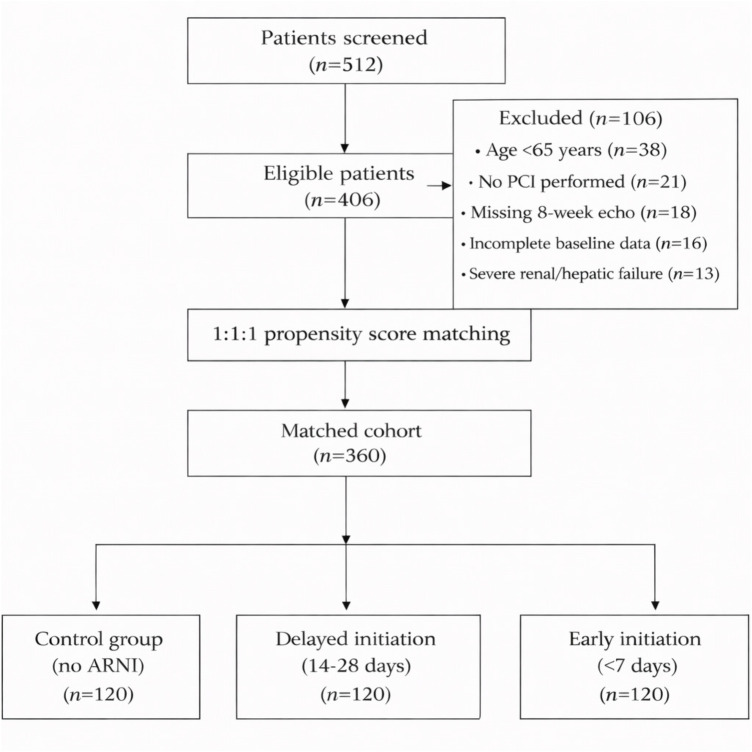
Patients selection.

**Figure 2:**
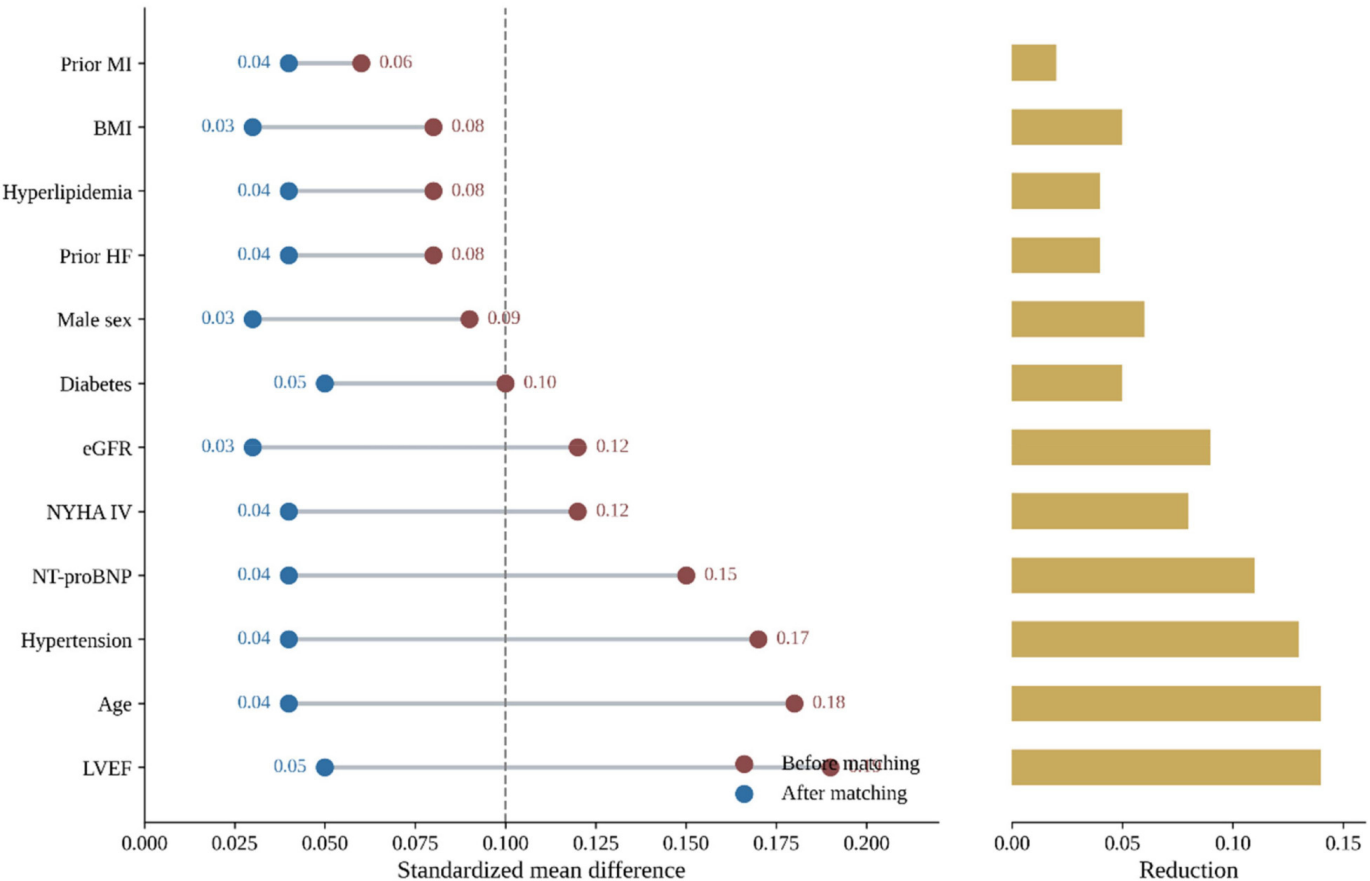
Love plot of standardized mean differences before and after propensity score matching.

A total of 512 elderly patients with left ventricular dysfunction who underwent successful PCI were initially screened. After applying exclusion criteria (Figure 1), 406 patients were eligible for the study. To minimize selection bias and emulate a comparative effectiveness trial, a 1:1:1 propensity score matching was performed, resulting in a final matched cohort of 360 patients, with 120 patients in each of the three groups: Control, Delayed Initiation, and Early Initiation. Following propensity score matching, baseline demographic characteristics, comorbidities, echocardiographic findings, and biomarker profiles were generally well balanced across the three groups (Table 1, Figure 2). As expected by design, ACEi/ARB use was markedly lower in the Early group (16.7%) compared with the Delayed (70.0%) and Control (70.8%) groups (SMD = 1.28). This imbalance is structural rather than confounding, as early sacubitril/valsartan initiation precludes concurrent ACEi/ARB therapy. However, it implies that the comparison between the Early group and the other two groups represents a comparison of two different treatment strategies (ARNI vs. ACEi/ARB) in addition to a comparison of timing. Consequently, any observed differences cannot be solely attributed to timing of initiation.

**Table 1:** Baseline characteristics of the matched cohort (*n* = 360).

Characteristic	Control (*n* = 120)	Delayed (*n* = 120)	Early (*n* = 120)	*P* value	SMD
Demographics					
Age, years, mean ± SD	72.1 ± 6.5	72.8 ± 7.0	72.3 ± 6.9	0.72	0.04
Age ≥75 years, *n* (%)	42 (35.0)	44 (36.7)	43 (35.8)	0.96	0.03
Male, *n* (%)	58 (48.3)	57 (47.5)	59 (49.2)	0.96	0.02
BMI, kg/m², mean ± SD	26.4 ± 3.2	26.1 ± 3.5	26.3 ± 3.3	0.78	0.05
Comorbidities, *n* (%)					
Hypertension	94 (78.3)	95 (79.2)	94 (78.3)	0.98	0.02
Diabetes mellitus	50 (41.7)	51 (42.5)	49 (40.8)	0.96	0.03
Dyslipidemia	88 (73.3)	89 (74.2)	87 (72.5)	0.95	0.04
Prior myocardial infarction	45 (37.5)	46 (38.3)	44 (36.7)	0.96	0.03
Prior heart failure	63 (52.5)	64 (53.3)	63 (52.5)	0.99	0.01
Atrial fibrillation	28 (23.3)	27 (22.5)	29 (24.2)	0.96	0.04
Chronic kidney disease (eGFR <60)	32 (26.7)	33 (27.5)	31 (25.8)	0.95	0.04
Medications at discharge, *n* (%)					
Aspirin	118 (98.3)	119 (99.2)	118 (98.3)	0.78	0.07
P2Y12 inhibitor	120 (100)	120 (100)	120 (100)	1	0
Beta-blocker	102 (85.0)	103 (85.8)	104 (86.7)	0.92	0.04
ACEi/ARB	85 (70.8)	84 (70.0)	20 (16.7)	<0.001	1.28
Statin	116 (96.7)	117 (97.5)	116 (96.7)	0.9	0.05
Baseline echocardiography					
LVEF, %, mean ± SD	41.5 ± 5.8	41.2 ± 5.9	41.4 ± 6.0	0.92	0.03
LVEF ≤40%, *n* (%)	58 (48.3)	59 (49.2)	58 (48.3)	0.99	0.02
LVEDD, mm, mean ± SD	54.2 ± 4.5	54.5 ± 4.8	54.0 ± 4.6	0.7	0.06
LVESD, mm, mean ± SD	41.1 ± 4.2	41.4 ± 4.5	40.9 ± 4.3	0.65	0.07
LA diameter, mm, mean ± SD	42.5 ± 3.8	42.8 ± 4.0	42.3 ± 3.9	0.6	0.08
Baseline biomarkers, median (IQR)					
hs-CRP, mg/L	8.5 (5.2–12.1)	8.1 (5.0–11.8)	8.3 (5.1–12.0)	0.85	0.05
IL-6, pg/mL	6.2 (4.1–9.0)	6.0 (3.9–8.8)	6.1 (4.0–8.9)	0.88	0.04
TNF-α, pg/mL	5.5 (3.8–7.9)	5.4 (3.7–7.8)	5.5 (3.8–7.8)	0.92	0.03
ICAM-1, ng/mL	320 (280–380)	315 (275–375)	318 (278–378)	0.82	0.06
NT-proBNP, pg/mL	850 (620–1,150)	820 (600–1,120)	840 (610–1,140)	0.78	0.06
cTnI, ng/mL	0.25 (0.18–0.35)	0.24 (0.17–0.34)	0.25 (0.18–0.36)	0.82	0.04
CK-MB, ng/mL	3.2 (2.4–4.5)	3.1 (2.3–4.4)	3.2 (2.4–4.6)	0.85	0.05
LDH, U/L	210 (185–245)	208 (182–242)	211 (186–246)	0.79	0.06

ACEi, angiotensin-converting enzyme inhibitor; ARB, angiotensin receptor blocker; BMI, body mass index; CK-MB, creatine kinase-MB; cTnI, cardiac troponin I; eGFR, estimated glomerular filtration rate; hs-CRP, high-sensitivity C-reactive protein; ICAM-1, intercellular adhesion molecule-1; IL-6, interleukin-6; IQR, interquartile range; LA, left atrium; LDH, lactate dehydrogenase; LVEDD, left ventricular end-diastolic diameter; LVEF, left ventricular ejection fraction; LVESD, left ventricular end-systolic diameter; NT-proBNP, N-terminal pro-brain natriuretic peptide; SD, standard deviation; SMD, standardized mean difference; TNF-α, tumor necrosis factor-alpha.

### 3.2 Association with 12-month clinical outcomes

**Figure 3:**
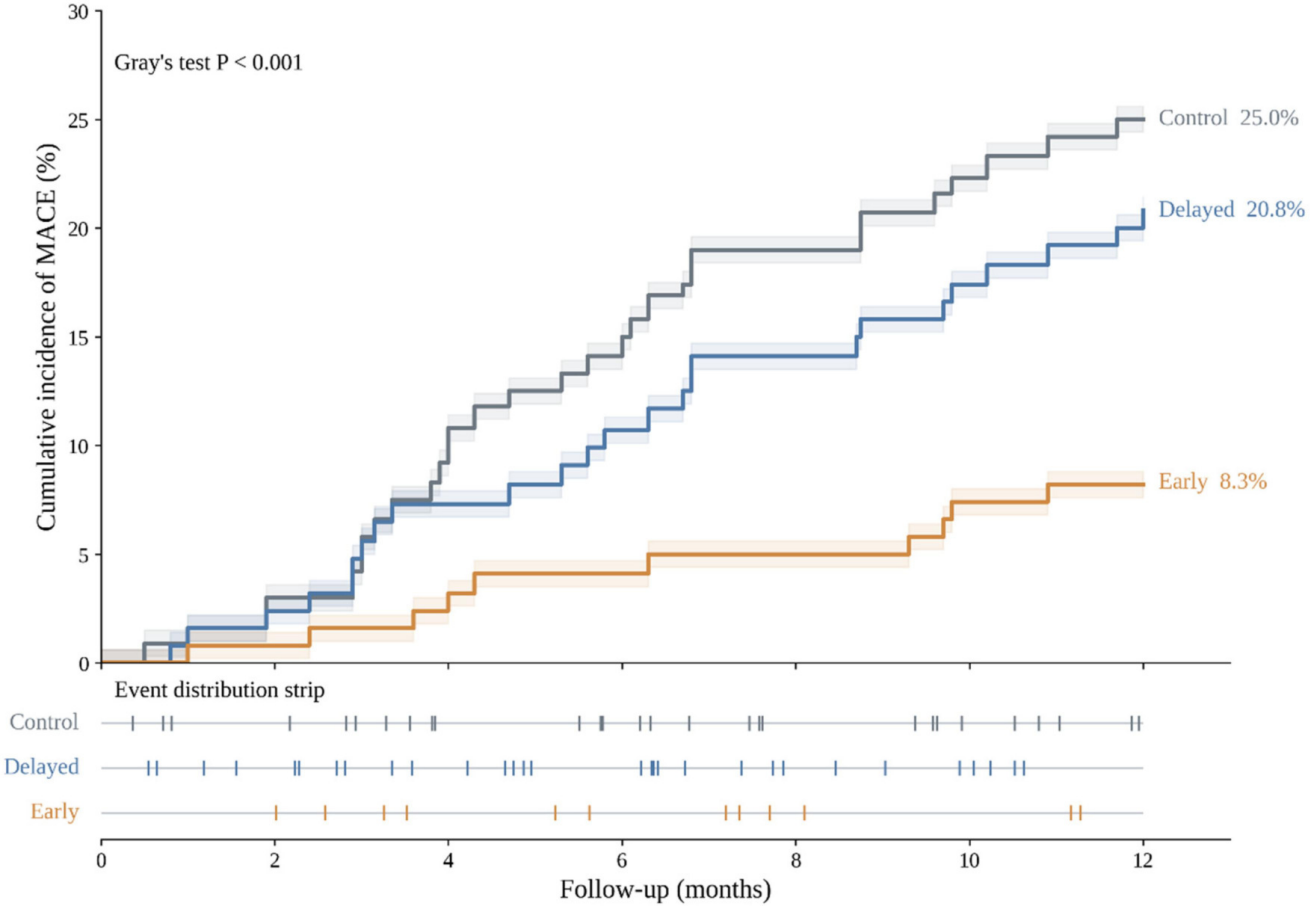
Cumulative incidence of MACE (competing risk analysis).

**Figure 4:**
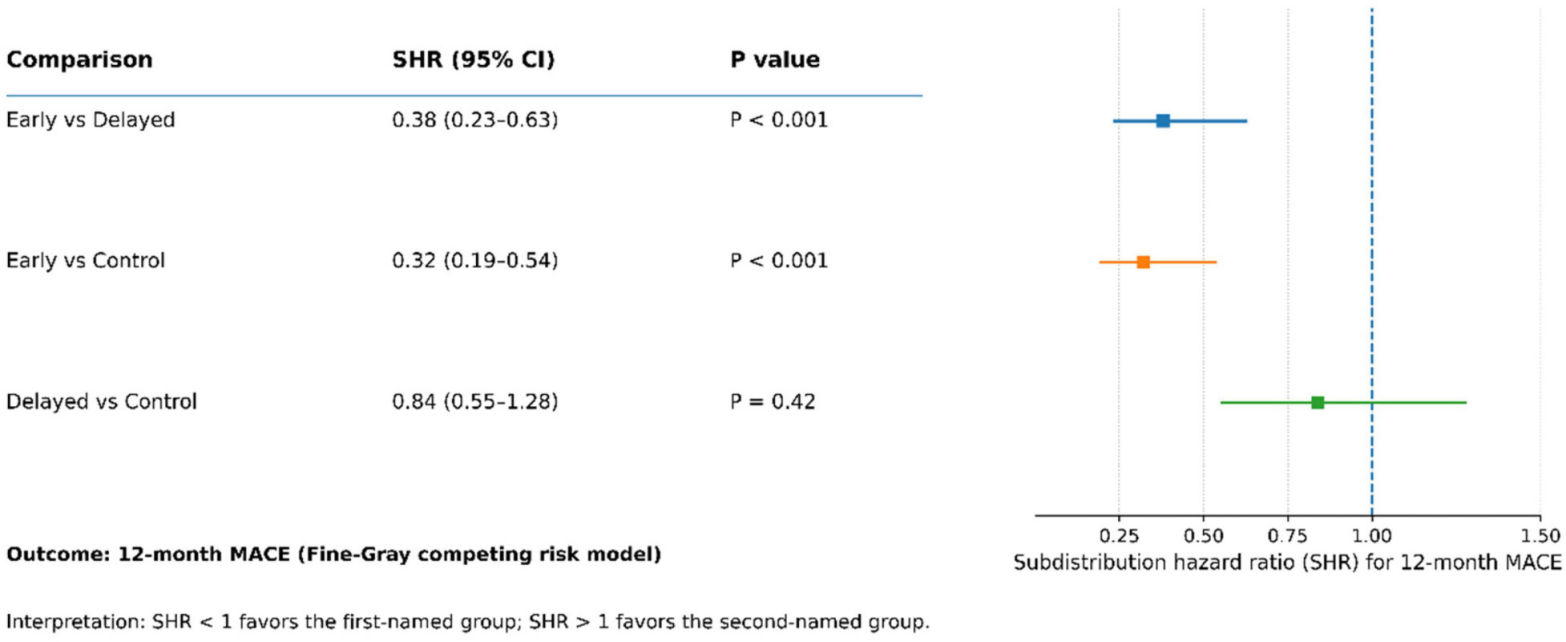
Forest plot of fine-gray models for MACE.

Over a median follow-up of 12 months, the cumulative incidence of MACE (accounting for non-cardiovascular death as a competing risk) was 8.3% (10 events) in the Early group, 20.8% (25 events) in the Delayed group, and 25.0% (30 events) in the Control group (Gray's test *P* < 0.001) (Table 2). Direct pairwise comparison showed that the Early group had a significantly lower MACE incidence than the Delayed group (8.3% vs. 20.8%, Gray's test *P* < 0.01), whereas the Delayed group did not differ significantly from the Control group (20.8% vs. 25.0%, Gray's test *P* = 0.42) (Figure 3). In the Fine-Gray competing risks regression model, early initiation was associated with a lower subdistribution hazard of MACE compared with delayed initiation (SHR = 0.38; 95% CI: 0.23–0.63; *P* < 0.001) and compared with the control group (SHR = 0.32; 95% CI: 0.19–0.54; *P* < 0.001). However, these cumulative incidence estimates may be affected by immortal time bias, as patients in the delayed group were required to survive event-free until day 14 to be classified as such. Consistent with the pairwise comparison findings, no significant difference in MACE risk was observed between the delayed and control groups (SHR = 0.84; 95% CI: 0.55–1.28; *P* = 0.42). (Figure 4).

**Table 2:** Clinical outcomes at 12 months.

Outcome	Control (*n* = 120)	Delayed (*n* = 120)	Early (*n* = 120)	*P* value
MACE, *n* (%)	30 (25.0)	25 (20.8)	10 (8.3)	<0.001
Cardiovascular death	5 (4.2)	4 (3.3)	1 (0.8)	0.25
Non-fatal MI	8 (6.7)	7 (5.8)	3 (2.5)	0.28
Unplanned revascularization	12 (10.0)	10 (8.3)	4 (3.3)	0.11
Hospitalization for HF	9 (7.5)	8 (6.7)	3 (2.5)	0.15
Stroke	4 (3.3)	3 (2.5)	1 (0.8)	0.40
Non-cardiovascular death, *n* (%)	2 (1.7)	3 (2.5)	1 (0.8)	0.60
Safety events, *n* (%)				
Symptomatic hypotension	6 (5.0)	7 (5.8)	8 (6.7)	0.86
Renal impairment	5 (4.2)	6 (5.0)	5 (4.2)	0.94
Bradycardia (HR <50 bpm)	4 (3.3)	5 (4.2)	6 (5.0)	0.82
Hyperkalemia (K + >5.5 mmol/L)	3 (2.5)	4 (3.3)	3 (2.5)	0.90
Angioedema	0 (0)	0 (0)	0 (0)	1．00

The sensitivity analyses yielded findings consistent with the primary analysis (Supplementary Tables S1, S2). In the 14-day landmark analysis, early initiation remained associated with a lower risk of MACE compared with delayed initiation (SHR = 0.42; 95% CI: 0.25–0.71) and control (SHR = 0.36; 95% CI: 0.21–0.62). In the time-dependent Cox model, sacubitril/valsartan use (as a time-varying exposure) was associated with a significantly lower risk of MACE compared with no use (HR = 0.47; 95% CI: 0.31–0.71). These results suggest that immortal time bias did not fully account for the observed associations.

### 3.3 Secondary mechanistic outcomes: echocardiographic myocardial recovery

At 8-week follow-up, all three groups showed improvements in echocardiographic parameters, but the degree of recovery differed significantly across groups (Table 3). Pairwise comparisons (Bonferroni-corrected) demonstrated that the Early Initiation group had significantly greater improvements than both the Control and Delayed groups for all parameters. The increase in LVEF was significantly larger in the Early group than in the Delayed group (*Δ*LVEF: 12.5% vs. 8.1%, *P* < 0.05), as was the reduction in LVEDD (−6.5 mm vs. −4.0 mm, *P* < 0.05) and LVESD (−7.1 mm vs. −4.2 mm, *P* < 0.05). Improvements in cardiac index also favored early over delayed initiation (*Δ*CI: 0.5 vs. 0.3 L/min/m², *P* < 0.05). No significant differences were observed between the Delayed and Control groups for any echocardiographic parameter after Bonferroni correction. Pairwise comparisons consistently showed that early initiation was superior to delayed initiation, whereas no significant differences were observed between delayed initiation and the control group across all echocardiographic parameters.

**Table 3:** Echocardiographic parameters at baseline and 8-week follow-up.

Parameter	Control (*n* = 120)	Delayed (*n* = 120)	Early (*n* = 120)	*P* (ANCOVA)
LVEF, %				<0.001
Baseline	41.5 ± 5.8	41.2 ± 5.9	41.4 ± 6.0	
8 weeks	46.7 ± 6.2	49.3 ± 6.5	53.9 ± 6.1	
*Δ*LVEF	5.2 ± 3.0	8.1 ± 3.5^a^	12.5 ± 4.2^a^^,^^b^	
LVEDD, mm				<0.001
Baseline	54.2 ± 4.5	54.5 ± 4.8	54.0 ± 4.6	
8 weeks	51.7 ± 4.2	50.5 ± 4.4	47.5 ± 4.1	
*Δ*LVEDD	−2.5 ± 1.5	−4.0 ± 1.8^a^	−6.5 ± 2.1^a^^,^^b^	
LVESD, mm				<0.001
Baseline	41.1 ± 4.2	41.4 ± 4.5	40.9 ± 4.3	
8 weeks	38.9 ± 4.0	37.2 ± 4.1	33.8 ± 3.8	
*Δ*LVESD	−2.2 ± 1.3	−4.2 ± 1.6^a^	−7.1 ± 2.0^a^^,^^b^	
Cardiac Index, L/min/m²				<0.001
Baseline	2.4 ± 0.3	2.4 ± 0.3	2.4 ± 0.3	
8 weeks	2.5 ± 0.3	2.7 ± 0.3	2.9 ± 0.4	
*Δ*Cardiac Index	0.1 ± 0.1	0.3 ± 0.1^a^	0.5 ± 0.2^a^^,^^b^	

a indicates *P* < 0.05 vs. Control;.

b indicates *P* < 0.05 vs. Delayed. Values with both symbols are significantly different from both groups.

### 3.4 Secondary mechanistic outcomes: changes in key biomarkers

Early initiation was associated with more favorable biomarker changes at 8 weeks (Tables 4, 5). Pairwise comparisons (Bonferroni-corrected) revealed that the Early group achieved significantly greater median percentage reductions than the Delayed group for the representative biomarkers hs-CRP (−58.2% vs. −41.2%, *P* < 0.05) and NT-proBNP (−65.5% vs. −48.3%, *P* < 0.05). Similar patterns were observed for IL-6, TNF-α, ICAM-1, cTnI, CK-MB, and LDH, where early initiation was associated with significantly larger reductions compared with delayed initiation (all *P* < 0.05). In contrast, reductions in the Delayed group did not differ significantly from those in the Control group for most biomarkers after correction for multiple comparisons. These findings support a timing-dependent association, with very early initiation showing the most favorable biomarker response. Given the correlation among these biomarkers and the observational design, these results should be interpreted as supportive evidence of a broader biological response pattern rather than as multiple independent confirmatory findings. In the main text, hs-CRP and NT-proBNP are highlighted as representative biomarkers, while the remaining markers are presented as supportive secondary biomarker data showing broadly concordant trends.

**Table 4:** Inflammatory biomarkers at baseline and 8 weeks.

Biomarker	Control (*n* = 120)	Delayed (*n* = 120)	Early (*n* = 120)	*P* (Kruskal–Wallis)
hs-CRP, mg/L				<0.001
Baseline	8.5 (5.2–12.1)	8.1 (5.0–11.8)	8.3 (5.1–12.0)	
8 weeks	7.1 (4.3–10.2)	4.5 (2.8–7.0)	2.9 (1.8–4.5)	
*Δ*%	−15.6 (−22.4 to −9.5)	−41.2 (−48.5 to −35.1) ^a^	−58.2 (−65.1 to −50.2) ^a^^,^^b^	
IL-6, pg/mL				<0.001
Baseline	6.2 (4.1–9.0)	6.0 (3.9–8.8)	6.1 (4.0–8.9)	
8 weeks	5.3 (3.5–7.8)	3.5 (2.4–5.2)	2.4 (1.6–3.5)	
*Δ*%	−13.5 (−19.2 to −8.0)	−39.8 (−46.0 to −33.5) ^a^	−58.5 (−65.2 to −51.0) ^a^^,^^b^	
TNF-α, pg/mL				<0.001
Baseline	5.5 (3.8–7.9)	5.4 (3.7–7.8)	5.5 (3.8–7.8)	
8 weeks	4.9 (3.3–7.0)	3.5 (2.5–5.0)	2.4 (1.7–3.4)	
*Δ*%	−11.8 (−17.5 to −6.5)	−34.5 (−40.8 to −28.0) ^a^	−55.2 (−62.0 to −48.1) ^a^^,^^b^	
ICAM-1, ng/mL				<0.001
Baseline	320 (280–380)	315 (275–375)	318 (278–378)	
8 weeks	295 (260–350)	245 (210–290)	195 (170–225)	
*Δ*%	−8.2 (−12.5 to −4.0)	−21.5 (−26.8 to −16.2) ^a^	−38.2 (−44.0 to −32.5) ^a^^,^^b^	

a indicates *P* < 0.05 vs. Control;.

b indicates *P* < 0.05 vs. Delayed. Values with both symbols are significantly different from both groups.

**Table 5:** Cardiac injury and neurohormonal biomarkers at baseline and 8 weeks.

Biomarker	Control (*n* = 120)	Delayed (*n* = 120)	Early (*n* = 120)	*P* (Kruskal–Wallis)
NT-proBNP, pg/mL				<0.001
Baseline	850 (620–1,150)	820 (600–1,120)	840 (610–1,140)	
8 weeks	680 (480–920)	420 (300–590)	280 (190–400)	
*Δ*%	−20.5 (−27.8 to −14.1)	−48.3 (−55.0 to −41.2) ^a^	−65.5 (−72.1 to −58.2) ^a^^,^^b^	
cTnI, ng/mL				<0.001
Baseline	0.25 (0.18–0.35)	0.24 (0.17–0.34)	0.25 (0.18–0.36)	
8 weeks	0.20 (0.14–0.28)	0.13 (0.09–0.18)	0.09 (0.06–0.12)	
*Δ*%	−18.5 (−24.0 to −13.0)	−44.2 (−50.5 to −38.0) ^a^	−61.5 (−68.0 to −55.0)^a^^,^^b^	
CK-MB, ng/mL				<0.001
Baseline	3.2 (2.4–4.5)	3.1 (2.3–4.4)	3.2 (2.4–4.6)	
8 weeks	2.6 (2.0–3.6)	1.8 (1.3–2.5)	1.3 (0.9–1.8)	
*Δ*%	−16.5 (−22.0 to −11.0)	−42.0 (−48.5 to −35.5) ^a^	−59.0 (−66.0 to −52.0) ^a^^,^^b^	
LDH, U/L				<0.001
Baseline	210 (185–245)	208 (182–242)	211 (186–246)	
8 weeks	192 (168–225)	162 (142–190)	135 (118–155)	
*Δ*%	−8.8 (−13.5 to −4.5)	−22.0 (−27.5 to −16.8) ^a^	−35.5 (−41.0 to −30.0) ^a^^,^^b^	

a indicates *P* < 0.05 vs. Control;.

b indicates *P* < 0.05 vs. Delayed. Values with both symbols are significantly different from both groups.

### 3.5 Exploratory subgroup analyses

**Figure 5:**
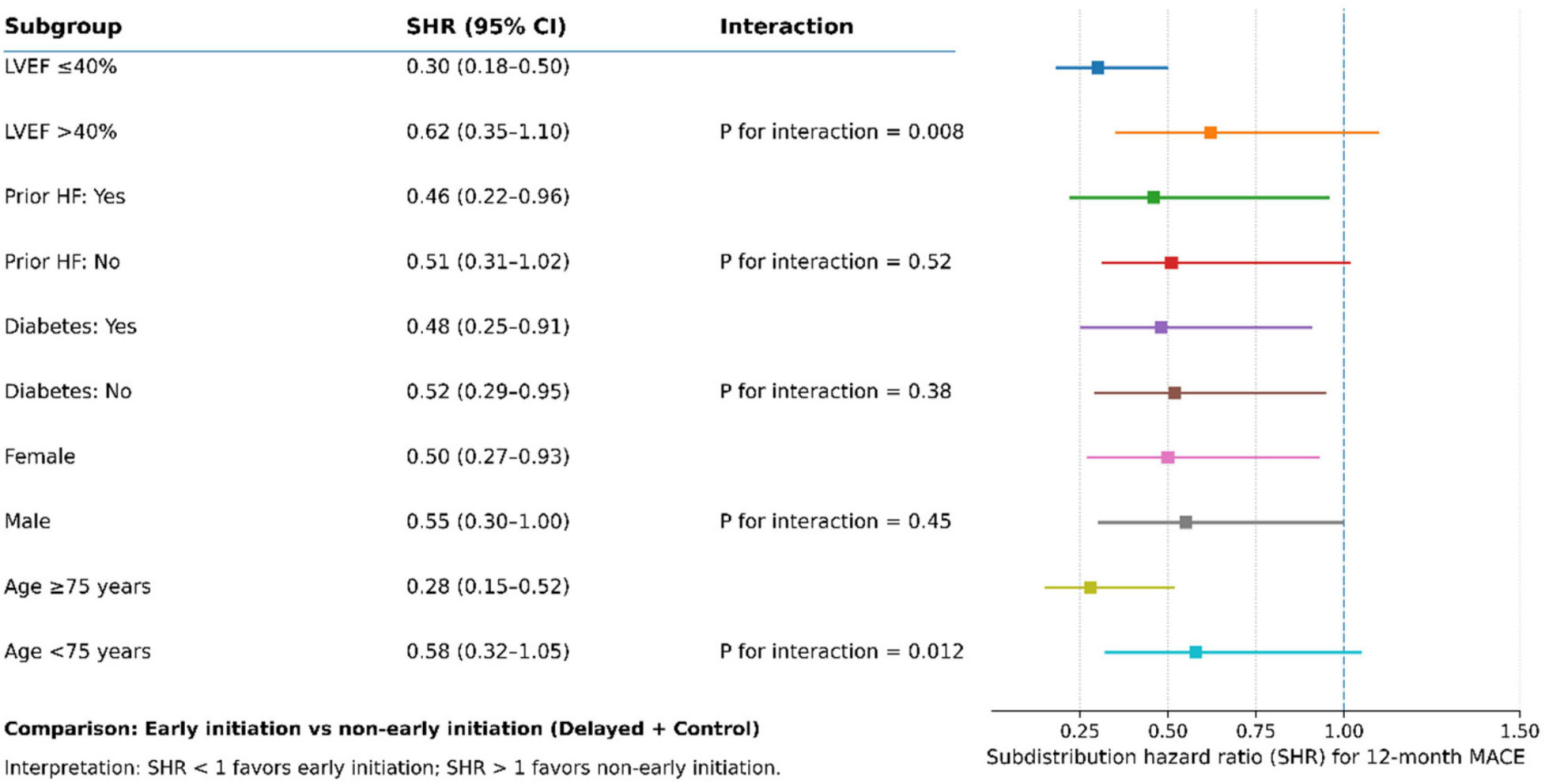
Subgroup analysis for MACE risk (early vs. Non-Early).

For subgroup analyses, the delayed and control groups were combined to increase statistical power and simplify interpretation, whereas pairwise comparisons are presented separately in Figure 4. To further assess the robustness of the findings, subgroup analyses were conducted across key clinical variables. Early initiation of ARNI was consistently associated with a reduced risk of 12-month MACE across all examined subgroups, with SHRs ranging approximately from 0.28 to 0.62 (Figure 5).

The magnitude of the association appeared more pronounced among patients aged ≥75 years and those with LVEF ≤40%, with significant interaction effects observed (*P* for interaction = 0.012 and 0.008, respectively). No significant interactions were identified for sex, diabetes status, or prior heart failure.

For these analyses, the delayed and control groups were combined as the reference category to enhance statistical power. Subgroup-specific estimates are detailed in Figure 5.

### 3.6 Exploratory integrated visualization

**Figure 6:**
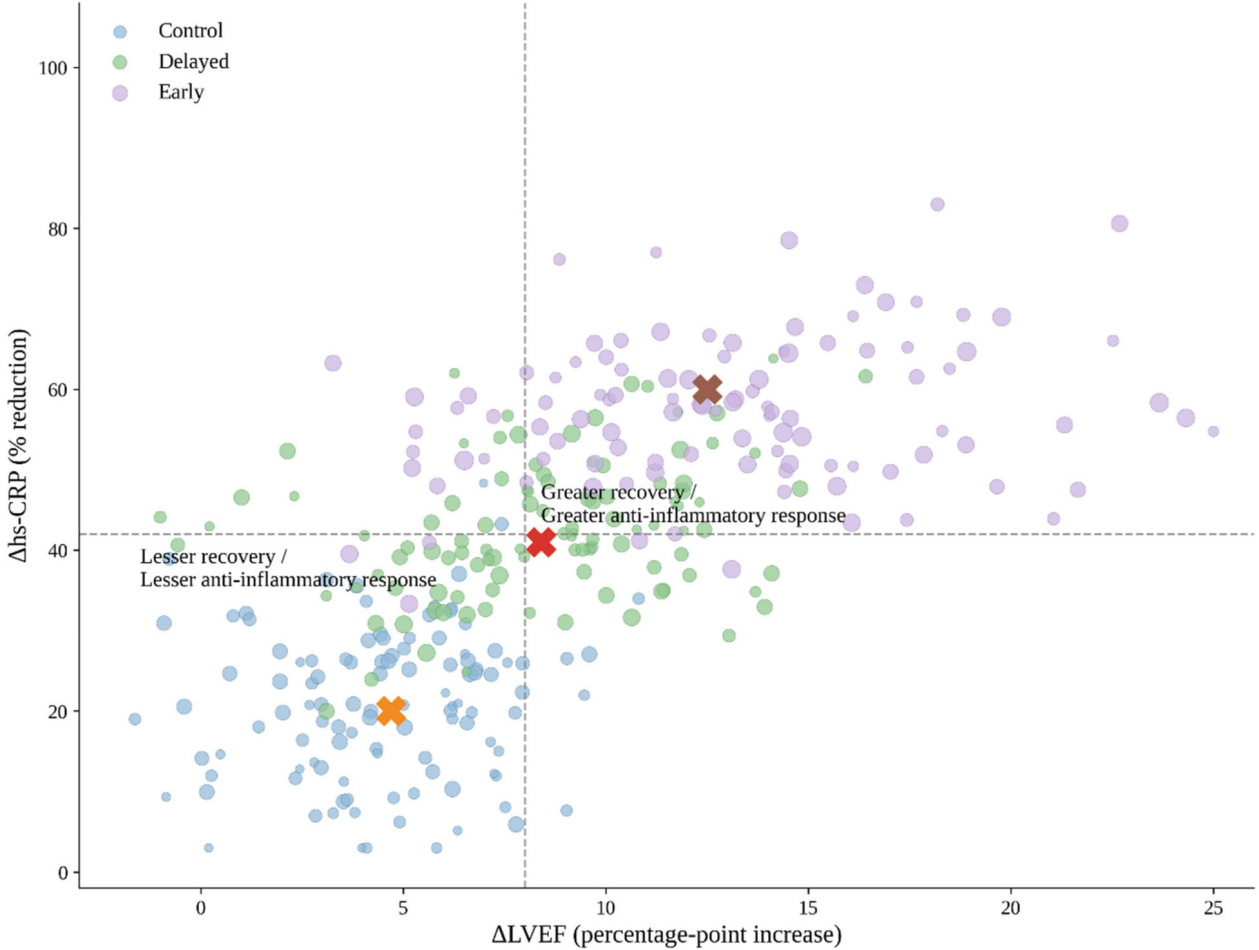
Integrated bubble plot of functional and biomarker changes.

An integrated analysis was performed to visualize the relationship between functional recovery and biomarker changes at the patient level. A bubble plot (Figure 6) depicts each patient in the matched cohort, with *Δ*LVEF on the *x*-axis, *Δ*hs-CRP on the *y*-axis, and bubble size representing the magnitude of NT-proBNP reduction. Patients in the Early group were more frequently distributed in the region characterized by greater LVEF improvement and larger reductions in hs-CRP and NT-proBNP. This pattern is consistent with a biologically plausible association between earlier initiation and more favorable concurrent changes in cardiac function and biomarker profiles. Nevertheless, due to the observational design and potential confounding, it should not be interpreted as evidence of a causal mechanistic pathway.

### 3.7 Exploratory safety outcomes

The incidence of adverse events was low and comparable across the three groups. There were no significant differences in the occurrence of symptomatic hypotension, renal impairment, bradycardia, or hyperkalemia. No cases of angioedema were reported during the study period. Adverse events were infrequent and numerically similar across groups. Although no clear excess safety signal was observed with early initiation in this matched cohort, the study was not powered to detect modest differences in individual safety outcomes.

## 4 Discussion

In this propensity score-matched retrospective cohort of elderly patients with left ventricular dysfunction after PCI, earlier initiation of sacubitril/valsartan was associated with more favorable short-term changes in echocardiographic parameters and biomarkers, as well as a lower 12-month risk of MACE, compared with delayed initiation or no sacubitril/valsartan. Importantly, the pattern of association appeared to vary according to treatment timing, with the most favorable profile consistently observed in the early initiation group. In exploratory subgroup analyses, these associations appeared more pronounced among patients aged ≥75 years and those with baseline LVEF ≤40%. However, because of the structural imbalance in background RAAS inhibitor therapy (ACEi/ARB vs. ARNI) between the early group and the other two groups, our findings should be interpreted as reflecting the combined effect of both earlier initiation and switching to ARNI-based therapy, rather than timing alone. Furthermore, potential immortal time bias in the delayed group may have influenced the effect estimates. Therefore, while our observations suggest a potential association, they should be viewed as hypothesis-generating and interpreted with substantial caution.

Our findings are broadly consistent with prior studies suggesting potential benefits of sacubitril/valsartan in post-myocardial infarction or post-PCI populations. Zhang et al. (8) reported that, among patients with STEMI undergoing primary PCI, sacubitril/valsartan initiated within 24 h was associated with better early cardiac functional recovery and lower NT-proBNP levels than conventional ACEI therapy. Likewise, Liu et al. (9) observed improved cardiac function and reverse remodeling in ACS patients with reduced LVEF treated with sacubitril/valsartan. In addition, meta-analyses by Zhang et al. (10) and Zhao et al. (11) suggested that early initiation of sacubitril/valsartan after acute myocardial infarction may be associated with improved cardiac function and lower rates of adverse cardiovascular events. Our study extends this literature by focusing specifically on an elderly cohort with left ventricular dysfunction after PCI and by comparing very early initiation with delayed initiation as separate exposure strategies. Rather than addressing only whether sacubitril/valsartan may be beneficial, our analysis suggests that the timing of initiation may be associated with different degrees of apparent benefit.

The observed differences in echocardiographic recovery are also biologically plausible. Post-infarction ventricular remodeling is a dynamic process driven by neurohormonal activation, inflammation, and structural myocardial changes (5, 13, 16). In this context, earlier neurohormonal modulation may theoretically be more effective during the early phase of post-ischemic remodeling than later treatment initiation. In our study, the early initiation group showed greater improvement in LVEF and cardiac index, together with larger reductions in LVEDD and LVESD, than the delayed and control groups. These findings are directionally consistent with previous reports indicating that sacubitril/valsartan may favorably influence reverse remodeling (12, 14, 18). However, because our study was observational and treatment allocation was not randomized, these differences should be interpreted as associations rather than definitive evidence that early initiation directly caused superior myocardial recovery.

A similar degree of caution is warranted when interpreting the biomarker findings. Patients in the early initiation group showed larger reductions in inflammatory, myocardial injury, and neurohormonal markers, including hs-CRP, IL-6, TNF-α, NT-proBNP, cTnI, CK-MB, and LDH. These patterns are broadly in line with previous reports. Pang et al. (15) found that sacubitril/valsartan-based treatment was associated with reductions in NT-proBNP and several inflammatory markers in elderly cardiovascular patients, while experimental work by Gan et al. (17) suggested that sacubitril/valsartan may attenuate inflammatory signaling and adverse remodeling pathways. In addition, mechanistic reviews have highlighted potential effects of sacubitril/valsartan on myocardial fibrosis, oxidative stress, and neurohormonal activation (19, 20). Nevertheless, the present biomarker analyses should be viewed as supportive rather than independently confirmatory. The markers assessed are biologically related and may reflect overlapping dimensions of disease activity and recovery. Accordingly, the concordant biomarker changes observed here are best interpreted as consistent with a broader pattern of more favorable recovery in the early initiation group, rather than as proof of a specific mechanistic pathway.

A clinically relevant aspect of the present study is the suggestion that any apparent advantage of early initiation may not be uniform across all elderly patients. In exploratory subgroup analyses, the association between early initiation and lower 12-month MACE appeared stronger in patients aged ≥75 years and in those with baseline LVEF ≤40%. These findings are clinically plausible, as such patients are likely to have greater baseline vulnerability to adverse remodeling and recurrent cardiovascular events (3–5). However, subgroup analyses in observational studies are inherently susceptible to limited power, residual confounding, and chance findings. Therefore, these interaction signals should be considered hypothesis-generating and should not be interpreted as establishing definitive treatment-effect heterogeneity. Their primary value lies in helping to identify patient groups that may warrant focused evaluation in future prospective studies.

The clinical outcome findings are also broadly consistent with the existing literature. In our matched cohort, the early initiation group had a lower cumulative incidence of MACE than the delayed and control groups, whereas the delayed group did not differ significantly from the control group. This pattern is directionally consistent with prior evidence suggesting that sacubitril/valsartan may reduce adverse cardiovascular events after myocardial infarction (10, 11, 21). At the same time, the magnitude of association observed in our study should be interpreted with caution. Even after propensity score matching, residual confounding by factors such as frailty, hemodynamic stability, clinician treatment preference, medication adherence, and post-discharge care intensity cannot be excluded. It is therefore possible that patients selected for very early initiation differed in ways not fully captured by the measured covariates. For this reason, our findings should be framed as supporting an association between earlier initiation and better outcomes, rather than demonstrating a causal benefit of early treatment.

With respect to tolerability, we did not observe a clear excess of adverse events in the early initiation group. Rates of symptomatic hypotension, renal impairment, bradycardia, and hyperkalemia were low and comparable across groups. These observations are generally consistent with prior safety data for sacubitril/valsartan in both trial and real-world settings (22–24). Tsutsui et al. (25) also showed that although the overall efficacy and safety profile of sacubitril/valsartan was acceptable across blood pressure strata, hypotension-related events may be more frequent in patients with lower baseline systolic blood pressure. In our cohort, baseline clinical characteristics were generally balanced after matching, which may partly explain the absence of an obvious safety signal. However, the present study was not powered to detect modest between-group differences in individual safety endpoints, and the safety findings should therefore be interpreted conservatively.

Several limitations of this study, spanning study design, confounding, bias, generalizability, and causal inference, warrant careful consideration.

First, confounding by indication is a major concern. Clinicians may have selectively offered very early ARNI (within 7 days) to patients with better hemodynamic stability, fewer comorbidities, or higher perceived likelihood of benefit, whereas those with complicated post-PCI courses (e.g., prolonged inotropic support, recurrent ischemia, or acute kidney injury) may have received delayed or no ARNI. Although propensity score matching adjusted for a wide range of measured confounders (including age, sex, BMI, comorbidities, baseline LVEF, and concomitant medications), residual confounding by unmeasured variables cannot be excluded. Such unmeasured factors include frailty, nutritional status, in-hospital complications (e.g., cardiogenic shock, malignant arrhythmias), socioeconomic status, health literacy, medication adherence, and the treating physician's preference for early ARNI based on subtle clinical cues not captured in electronic medical records.

Second, the structural imbalance in background renin-angiotensin-aldosterone system inhibitor therapy fundamentally limits interpretation. After matching, ACEi/ARB use was 70.8% in the control group, 70.0% in the delayed group, and only 16.7% in the early group (SMD = 1.28, far exceeding the threshold for acceptable balance). This imbalance is not a confounding error but a direct consequence of the exposure definition: patients assigned to early sacubitril/valsartan were not maintained on conventional ACEi/ARB therapy during the same period. Consequently, the comparison between the early group and the other two groups confounds two distinct factors: (1) treatment type (ARNI vs. ACEi/ARB) and (2) treatment timing (early vs. delayed/none). The observed benefits in echocardiographic recovery, biomarker reduction, and MACE risk could be partially or fully attributable to the intrinsic superiority of ARNI over ACEi/ARB (as established in the PARADIGM-HF trial), rather than to earlier initiation *per se*. With the current observational design, we cannot disentangle these two effects. Any interpretation that attributes the findings solely to “timing” is therefore an overstatement.

Third, immortal time bias may have influenced the effect estimates, particularly for the delayed initiation group. By definition, patients in the delayed group had to survive event-free from the index PCI until day 14 to receive treatment. This creates a guaranteed event-free window that does not exist for the control group, who were never exposed to ARNI. As a result, the cumulative incidence of MACE in the delayed group may be artificially lowered, potentially explaining why we observed no significant difference between the delayed and control groups (SHR = 0.84; 95% CI: 0.55–1.28; *P* = 0.42). To address this bias, we performed two prespecified sensitivity analyses: (1) a 14-day landmark analysis that excluded all patients who experienced MACE or non-cardiovascular death within the first 14 days (early group: 1 excluded; delayed: 2; control: 3), and (2) a time-dependent Cox regression model that treated sacubitril/valsartan initiation as a time-varying covariate, thus completely avoiding fixed time-zero assignment. Both analyses yielded findings consistent with the primary analysis: in the landmark analysis, early initiation remained associated with a lower risk of MACE compared with delayed initiation (SHR = 0.42; 95% CI: 0.25–0.71) and control (SHR = 0.36; 95% CI: 0.21–0.62); in the time-dependent model, any ARNI use (time-varying) was associated with a lower risk compared with no use (HR = 0.47; 95% CI: 0.31–0.71). These sensitivity analyses suggest that immortal time bias did not fully account for the observed associations, but residual bias cannot be completely excluded.

Fourth, the sample size is moderate, and the number of clinical events is limited. After matching, each group contained 120 patients, and the total number of MACE events was only 65 (10 in the early group, 25 in the delayed group, and 30 in the control group). For subgroup and interaction analyses (e.g., age ≥75 years, LVEF ≤40%), the event counts become even smaller, leading to wide confidence intervals and unstable estimates. The interaction *P* values (0.012 for age and 0.008 for LVEF) should be interpreted with extreme caution; they are exploratory and hypothesis-generating, not confirmatory. No adjustment for multiple comparisons was applied to secondary or exploratory outcomes, which increases the risk of false-positive findings.

Fifth, the follow-up duration for mechanistic outcomes is relatively short. Echocardiographic and biomarker reassessments were performed at approximately 8 weeks after PCI. While this time point captures early reverse remodeling, it may not fully capture the longer-term trajectory of ventricular remodeling (e.g., progressive fibrosis or late dilatation) that can occur over 6 to 12 months. Longer-term echocardiographic follow-up would be required to determine whether the early benefits are sustained, attenuated, or even amplified over time.

Sixth, treatment exposure was based on real-world clinical practice rather than a protocolized, randomized assignment. There was substantial variation in starting doses (e.g., 24/26 mg vs. 49/51 mg twice daily), dose titration schedules, treatment persistence (possible discontinuation due to side effects or cost), and adherence. Additionally, concurrent therapies such as beta-blockers (approximately 85% across groups) and mineralocorticoid receptor antagonists (not reported) may have differed in unmeasured ways. These real-world variations could have influenced the observed outcomes and introduce bias that cannot be fully adjusted for.

Seventh, the 12-month follow-up for clinical outcomes is relatively short. Cardiovascular events, including late heart failure hospitalizations and cardiovascular deaths, may continue to accrue beyond 1 year. Longer-term follow-up (e.g., 3–5 years) would provide a more complete picture of the clinical benefit–risk profile of early vs. delayed ARNI initiation.

Eighth, generalizability is limited by the single-center, retrospective design and specific inclusion/exclusion criteria. Our study was conducted at a single tertiary hospital in China, and all patients were of Asian ethnicity. The findings may not be generalizable to other institutions with different practice patterns, to non-Asian populations, or to healthcare systems with different resources and follow-up protocols. Furthermore, we excluded patients with eGFR <30 mL/min/1.73 m², severe hepatic impairment, or expected survival <12 months due to non-cardiovascular comorbidities; thus, our results do not apply to these higher-risk or contraindicated groups.

Finally, despite the use of propensity score matching and sensitivity analyses, this remains an observational study. Causality cannot be inferred. The possibility of residual confounding by unmeasured variables (e.g., frailty, hemodynamic trajectory in the first 48 h, detailed medication adherence) cannot be eliminated. Therefore, all findings presented here should be interpreted as hypothesis-generating only. Prospective randomized controlled trials that directly compare very early vs. delayed ARNI initiation (with both arms receiving ARNI, thereby isolating the timing effect) are required before any definitive conclusions about optimal treatment timing can be drawn.

Despite these limitations, the study has potential clinical relevance. In routine practice, the question is often not only whether sacubitril/valsartan should be used after PCI in patients with left ventricular dysfunction, but also when it may be most appropriate to initiate treatment, particularly in older and more vulnerable individuals. Our findings suggest that very early initiation may be associated with a more favorable recovery profile than delayed initiation, especially in patients at higher baseline risk. However, these observations should be considered preliminary and should not be overgeneralized to treatment recommendations without confirmation from prospective studies.

Future research should move beyond simple treatment-vs.-no-treatment comparisons and directly test whether timing of initiation modifies clinical benefit in elderly patients after PCI. Prospective multicenter studies, ideally with randomized or carefully protocolized designs, are needed to compare very early vs. delayed initiation strategies and to determine whether older age or greater baseline systolic dysfunction truly identifies patients with greater benefit. Additional mechanistic studies integrating imaging and biomarker profiling may also help clarify whether the observed associations reflect differential effects on post-infarction remodeling and inflammatory recovery (17, 18).

In conclusion, this propensity score-matched retrospective study of elderly patients with left ventricular dysfunction after PCI found that very early initiation of sacubitril/valsartan (≤7 days) was associated with more favorable short-term myocardial recovery and a lower 12-month risk of MACE compared with delayed initiation or no sacubitril/valsartan. However, these associations cannot be distinguished from the effects of ARNI vs. ACEi/ARB therapy, and potential immortal time bias may have influenced the results. The observed exploratory interaction signals in patients aged ≥75 years and those with baseline LVEF ≤40% are hypothesis-generating. At present, the hypothesis that early ARNI initiation after PCI provides incremental benefit beyond ARNI itself remains unproven. Prospective randomized trials directly comparing early vs. delayed ARNI initiation (with both groups receiving ARNI) are required before any definitive conclusions about optimal timing can be drawn.

## Data Availability

The raw data supporting the conclusions of this article will be made available by the authors, without undue reservation.
