# Supplementary material for: Heterogeneous benefit of early sacubitril/valsartan initiation after PCI in elderly patients with left ventricular dysfunction: myocardial recovery and mid-term cardiovascular outcomes
Source: Front Cardiovasc Med. 2026 May 29;13:1843561. doi: 10.3389/fcvm.2026.1843561 (PMC13260289; doi:10.3389/fcvm.2026.1843561)
Supplement: Supplementary file 1 [file Datasheet1.docx]

**Supplementary Table S1. 14‑Day Landmark Analysis for MACE (Excluding Events Within First 14 Days)**

| **Group** | **Patients Remaining (n)** | **MACE Events (n)** | **Cumulative Incidence (%)** | **Fine‑Gray SHR (95% CI)** | **P value** |
| --- | --- | --- | --- | --- | --- |
| Early initiation (≤7 days) | 119 | 9 | 7.6 | 1.00 (reference) | — |
| Delayed initiation (14–28 days) | 118 | 23 | 19.5 | 0.42 (0.25–0.71) | <0.001 |
| Control (no ARNI) | 117 | 27 | 23.1 | 0.36 (0.21–0.62) | <0.001 |

**Supplementary Table S2. Time‑Dependent Cox Regression for MACE (Sacubitril/Valsartan as Time‑Varying Exposure)**

| **Exposure** | **Number of Patients** | **Number of Events** | **HR (95% CI)** | **P value** |
| --- | --- | --- | --- | --- |
| Sacubitril/valsartan use (time‑dependent) | 240 (anytime use) | 35 | 0.47 (0.31–0.71) | <0.001 |
| No sacubitril/valsartan use (reference) | 120 | 30 | 1 | — |
